# Microbial community development on the surface of Hans and Werenskiold Glaciers (Svalbard, Arctic): a comparison

**DOI:** 10.1007/s00792-015-0764-z

**Published:** 2015-06-24

**Authors:** Jakub Grzesiak, Dorota Górniak, Aleksander Świątecki, Tamara Aleksandrzak-Piekarczyk, Katarzyna Szatraj, Marek K. Zdanowski

**Affiliations:** Department of Antarctic Biology, Institute of Biochemistry and Biophysics, Polish Academy of Sciences, Pawińskiego 5a, 02-106 Warsaw, Poland; Department of Microbiology, University of Warmia and Mazury, Oczapowskiego 1A, 10-719 Olsztyn, Poland; Department of Microbial Biochemistry, Institute of Biochemistry and Biophysics, Polish Academy of Sciences, Pawińskiego 5a, 02-106 Warsaw, Poland; Institute of Geophysics, Polish Academy of Sciences, Księcia Janusza 64, 01-452 Warsaw, Poland

**Keywords:** Tidewater glacier, Glacial ice, Cryoconite holes, Microbial community, Microbial abundance, Snow line, Photoautotroph

## Abstract

Surface ice and cryoconite holes of two types of polythermal Svalbard Glaciers (Hans Glacier—grounded tidewater glacier and Werenskiold Glacier—land-based valley glacier) were investigated in terms of chemical composition, microbial abundance and diversity. Gathered data served to describe supraglacial habitats and to compare microbe–environment interactions on those different type glaciers. Hans Glacier samples displayed elevated nutrient levels (DOC, nitrogen and seston) compared to Werenskiold Glacier. Adjacent tundra formations, bird nesting sites and marine aerosol were candidates for allochtonic enrichment sources. Microbial numbers were comparable on both glaciers, with surface ice containing cells in the range of 10^4^ mL^−1^ and cryoconite sediment 10^8^ g^−1^ dry weight. Denaturating gradient gel electrophoresis band-based clustering revealed differences between glaciers in terms of dominant bacterial taxa structure. Microbial community on Werenskiold Glacier benefited from the snow-released substances. On Hans Glacier, this effect was not as pronounced, affecting mainly the photoautotrophs. Over-fertilization of Hans Glacier surface was proposed as the major factor, desensitizing the microbial community to the snow melt event. Nitrogen emerged as a limiting factor in surface ice habitats, especially to Eukaryotic algae.

**Electronic supplementary material:**

The online version of this article (doi:10.1007/s00792-015-0764-z) contains supplementary material, which is available to authorized users.

## Introduction

Glaciers are described by the scientific community as highly dynamic systems, constantly balancing between ice mass accumulation (Meier 1973; Jansson 1999), and ablation determined by surface energy balance (Lang 1968; Hock 1998) and calving (Brown et al. 1982). However, biologically, glacial ice has been regarded for long to be inactive or in later years to act only as a life-entrapping medium, collecting and preserving deposited microorganisms, derived via atmospheric precipitation (Butinar et al. 2007). Recent studies have revealed that parts of a glacier with certain physical and chemical conditions can be a relatively favorable environment that supports active and diverse communities of not only micro- but also macrobiota (Hodson et al. 2008; Anesio and Laybourn-Parry 2012). The ablating glacier surface receives solar radiation, yielding liquid water essential for biological processes and is covered by debris particles to a varying degree (debris amount generally increases toward the glacier terminus) which act as a source of biogenic elements like nitrogen and phosphorus (Franzetti et al. 2013; Anesio et al. 2009; Kennedy 1993). The dark dust deposit, known as cryoconite in sufficient quantities, reduces ice surface albedo and accelerates melting. As the ice melts, a water-filled hole forms, into which the dark material sinks—a so-called cryoconite hole (Anesio et al. 2010). When the melt processes gradually take place during the season (surface melt of winter snow), they cause the snow line to recede upward the glacier and further ablation of exposed ice. Consequently, the ablation zone comes to resemble a rigid ice sponge saturated with water and enriched with mineral and organic wind-borne particles capable of supporting life. These organisms, however, have to struggle with intense UV radiation, freeze–thaw cycles, low pH, nutrient deficits and other damaging factors (Edwards et al. 2014).

To get more insight into the glacial biome, researchers compared glaciers on global and local scales, but only few comparisons between supraglacial microbial communities have been published (Cameron et al. 2012; Edwards et al. 2011, 2013; Porazinska et al. 2004). Cryoconite holes have been the major focus of these studies. Although differences between Arctic and Antarctic glaciers were clear and easy to explain (the authors assumed variations in carbon content), the ones between neighboring glaciers were also apparent but much more difficult to elucidate. Edwards et al. (2011, 2013) explored three Spitsbergen valley glaciers and found out that bacterial communities present in cryoconite holes show some differences between those glaciers, yet they harbor distinct lineages, unlike those that dwell in adjacent terrestrial habitats. Ridgelines between the studied glaciers, different meltwater drainage patterns and environmental pressure have been postulated to be responsible for the discrepancies between glacier communities. However, no other factors (chemical or physical) have been shown to be of influence. Porazińska et al. (2004) investigated a similar situation in Taylor Valley, Antarctica. These authors found that cryoconite holes differ from glacier to glacier not only by means of physical parameters, but also in terms of organism diversity, mainly in the quality of primary producers. Here, freshwater reservoirs and nitrogen-rich soils in the particular glaciers vicinity were postulated as possible biota and nutrient sources, but many occurring phenomena have not been explained.

The information on comparative glacial microbiology remains scarce, involving mainly valley glaciers. Here, we investigate and compare microbial communities and their development on two types of polythermal Svalbard Glaciers. Hans Glacier is a grounded tidewater glacier which flows into the fjord of Hornsund in southern Spitsbergen. Werenskiold Glacier is a land-based valley glacier, next to Hans Glacier, but flowing from east to west (Pälli et al. 2003). To our knowledge, a comparison of microbial communities in supraglacial habitats between a tidewater and a land-based valley glacier has never been presented. Furthermore, Hans Glacier has never been investigated as a microbial habitat, despite its deep glaciological description (Grabiec et al. 2012; Migała et al. 2006; Oerlemans et al. 2011) and extensive microbiological works on the neighboring Werenskiold Glacier throughout many years (Stibal et al. 2006, 2007, 2008, 2009; Kaštovská et al. 2007).

We hypothesize that glacier tongues, originating from the same ice cap, are influenced by their immediate environmental surroundings, which are reflected in microbial community development of their supraglacial habitats. We expect a more abundant and active microbial community to flourish on a glacier surface experiencing an intense enrichment in organic and inorganic biogenic substances due to the proximity to the ocean, bird nesting sites and tundra formations. To test this hypothesis, we compared the glaciers’ microbial communities in terms of abundance and diversity and investigated spatial variation on the ablation zone and environmental controls of microbial processes. Physico-chemical and biological data were analyzed through simple regression and principal component analysis to provide insight into the still limited knowledge regarding interactions between glaciers and their inhabitants.

## Materials and methods

### Sites and sampling

Hans and Werenskiold Glaciers are located on the north shore of the Hornsund Fiord at Spitsbergen Island (Svalbard Archipelago) in Arctic. Hans Glacier, a grounded tidewater glacier, has a surface of about 57 km^2^ and its bottom reaches 100 m below sea level. Maximum ice thickness was estimated to be 400 m. Werenskiold Glacier is a land-based valley glacier next to Hans Glacier. It occupies an area of 27.11 km^2^ with a maximum ice thickness of 235 ± 15 m (Pälli et al. 2003). An extensive, scarcely vegetated forefield stretches in front of this glacier for ca 4 km to the fjord shore with several proglacial kettle lakes (Kabala and Zapart 2012). Both these glaciers are separated from the neighboring tundra and river–lake ecosystems by tall lateral moraines and mountain ridges (Pälli et al. 2003). In the Hans Glacier vicinity, large nesting places of several bird species have been established (Jakubas et al. 2008).

**Fig. 1:**
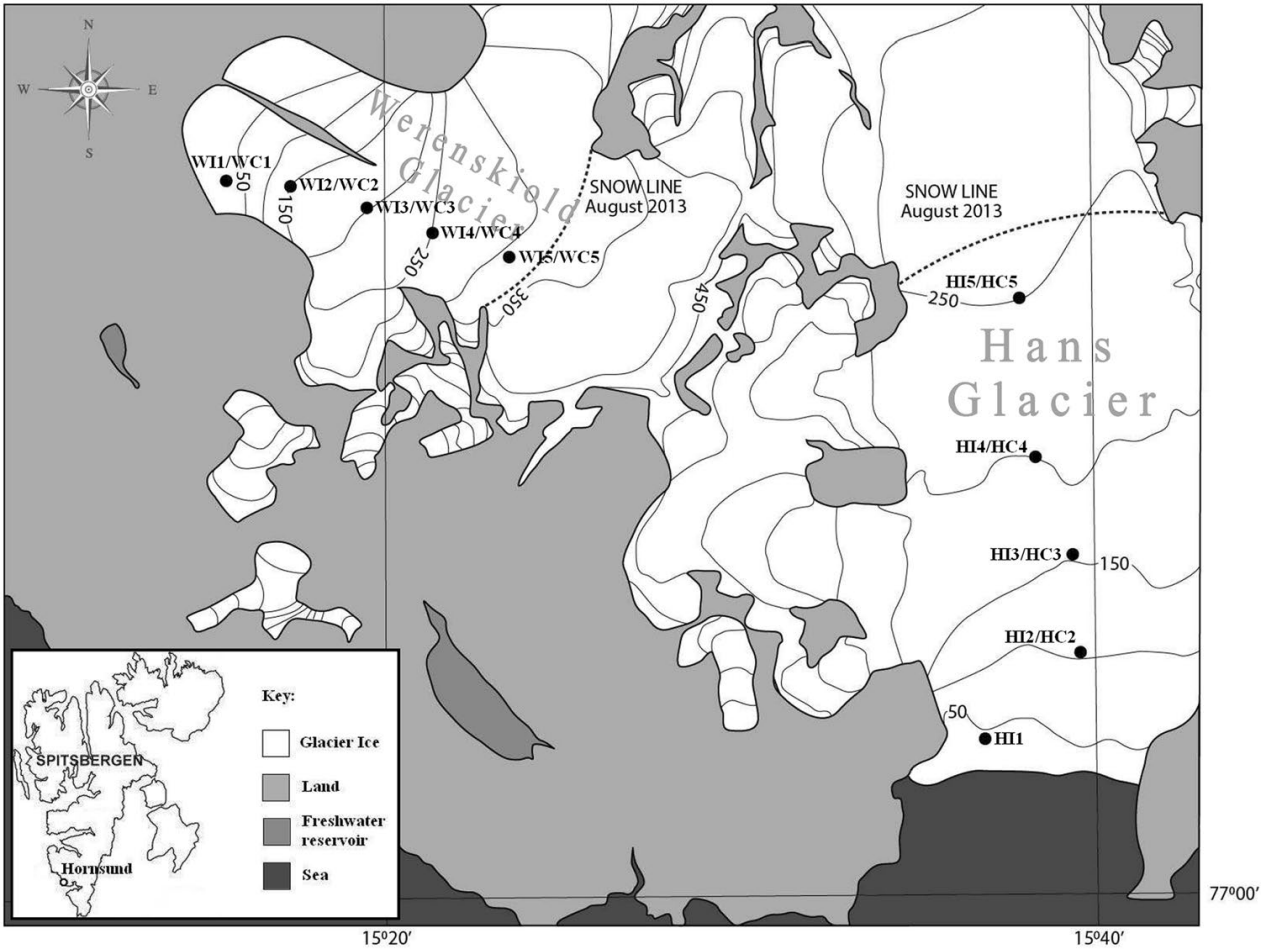
Location of sampling points on Hans and Werenskiold Glacier surface

Ice and cryoconite material were taken from 5 points on the glaciers surface in a transect running up the glacier, from glacial terminus area to the snow line at the top of the ablation zone (Fig. 1). The transect on Hans Glacier had a length of 5120 m and on Werenskiold Glacier—3420 m. Ice samples were termed HI (Hans Glacier) and WI (Werenskiold Glacier) with HI1/WI1 the first point on the ablation zone and HI5/WI5 the snow line point. Cryoconite samples were termed HC2 to HC5 for Hans Glacier and WC1 to WC5 for Werenskiold Glacier, each number indicating the same area as the surface ice samples (no cryoconite holes were found in HI1 area). Locations of the sampling points are presented in Table S1 (Supplementary Materials).

During August of 2013, ice from the glacier’s surface (approx. 20 cm) was crushed with an 70 % EtOH sterilized and deionized water-washed Tonar ice auger (158 cm long, 130 mm diameter), collected using sterile plastic spatulas and placed into sterile plastic bags. The crushed ice was gathered from 5 points per sampling site, in an area of 100 m^2^. Pooled cores totaled 3 kg per site. Cryoconite holes were drained of water and sediment with a 160 mL sterile plastic syringe, and the material was transported in 500 mL sterile bottles to a field laboratory and processed within 2 h. Five cryoconite holes per site were drained and pooled. A duplicate set of samples was stored at −20 °C and further research was conducted at the Department of Microbiology (University of Warmia and Mazury, Olsztyn, Poland) and Institute of Biochemistry and Biophysics of the Polish Academy of Sciences (Warsaw, Poland).

### Measurement of ice and cryoconite components

Chloride, sulfate, nitrate, sodium, potassium, calcium and magnesium ion concentrations were determined by high-pressure liquid chromatography (HPLC) in a Shimadzu Prominence modular HPLC model device. Phosphates, total phosphorus (after mineralization), total iron and silicate contents were measured in a Shimadzu UV 1601 spectrophotometer, while nitrates were measured on an Epoll-Eco 20 spectrophotometer (Standard Methods 1980; Hermanowicz et al. 1999). Total organic carbon (TOC), dissolved organic carbon (DOC), particulate organic carbon (POC) and total nitrogen contents were determined in a Shimadzu TOCV-CSH organic carbon analyzer with a TNM-1 total nitrogen analyzer attachment. Particulate content in water was determined by filtering the sample through a combusted Whatman GF/C glass fiber filter (1.2 µm pore size) which was then dried at 105^º^C; organic matter content was measured as that amount lost on ignition after combustion at 550^º^C. Chlorophyll *a* and pheophytin concentrations were calculated after the Lorenzen equation after extraction in acetone (at 4ºC) and spectrophotometry. A DOC spectrum was determined in a quartz cuvette at 260 nm in a Schimadzu UV-1601 bichannel scanning spectrophotometer (Shinichi et al. 2004). The spectrum was corrected against a demineralized water background. SUVA (specific UV absorbance) was calculated as the relative content of aromatic matter (=Abs_260_ × 1000 DOC^−1^).

### Sample preparation for microbiology

Ice samples were allowed to melt in a refrigerator (4 °C) before being processed for microbiological analyses. 1 ml of cryoconite–water mix was placed in a 2 ml sterile plastic Eppendorf type tube and centrifuged at 9000 rpm for 3 min in an MPW-52 microcentrifuge to separate the sediment from the water. After discarding the water part, the tube was weighted. 1 ml of melted, filter and heat sterilized melted glacier ice was added and the sediment was resuspended by vortexing the tube at 1000 rpm (Biosan type V-1 plus Personal Vortex) for 5 min. A duplicate test tube with sediment was used for dry weight (d.w.) determination after 24-h incubation at 65 °C in a dry box with circulating air (Dowgiałło 1975). Suspensions after vortexing were then stored in the refrigerator (4 °C) for 10–20 min to allow larger particles to settle. Aliquots of melted ice (1 mL, 0.5 mL and 0.1 mL) and cryoconite suspension dilutions (10^−1^, 10^−2^, 10^−3^; 0.1 mL each) were plated on R2A agar (Biocorp). Inoculated plates were incubated in darkness at 4 °C for 6 weeks. Sub-samples for total counts (TCs) were fixed with buffered formalin to a final concentration of 1 %. Additionally, 300 mL of melted ice was run through a 47 mm polycarbonate filter with 0.2 µm pore size. The filter was placed in a 50 mL sterile plastic cup and shaken with 20 mL of melted ice of the same origin point. The suspension was frozen for further analysis.

### Microbial abundance

Total counts (TC) were determined in 5 mL of melted glacier ice and 1 mL of 10^2^ diluted sediment suspension. TCs were determined by epifluorescence microscopy using 4, 6-di-amidino-2-phenylindole (DAPI) on black Nuclepore polycarbonate 0.2 µm pore size filters (Porter and Feig, 1980), under a Nikon E-200 microscope with a 100 W Hg lamp and 1009 CFI 60 oil immersion objective, with a digital DS Cooled Camera Head DS-5Mc-U1, and a filter block of wavelengths EX 330-380, DM 400, BA 420. Images of fields were analyzed in Nikon NIS Elements BR 2.30 and MultiScan v. 14.02 (computer scanning systems). A minimum of 400 cells in 20 fields per sample were counted automatically in the image analysis system. Average values of three measurements using three independently prepared filters were calculated. Dividing cells were counted according to Hagström et al. (1979). Photoautotrophs, including cyanobacteria and photoautotrophic eukaryotes, were counted under blue 450–490 nm (B-2A Nikon filter) and green 510–560 nm (G-2A Nikon filter) light excitation in an epifluorescence microscope (Putland and Rivkin 1999). Cyanobacteria were distinguished from photoautotrophic eukaryotes because of the former’s gold-yellow autofluorescence (Rassoulzadegan and Sheldon 1986). Culturable microorganisms abundance was performed by CFU enumeration after 42 days of incubation on R2A agar.

### Denaturating gradient gel electrophoresis analysis (DGGE)

The 500 mL water samples were vacuum filtered through a 0.2-µm pore-sized (ø 47 mm) white polycarbonate membrane filter (Millipore GTTP) mounted on a sterile bottle top filter holder (Nalgene). Total DNA was extracted from membranes with the use of an UltraClean™ Water DNA Isolation Kit (MoBio, Carlsbad, CA, USA) in accordance with the manufacturer’s protocol. DNA quality and yield were measured with a NanoVue™ spectrophotometer (GE Healthcare Life Science, Germany). The extracted DNA was stored at −20 °C until further use. DNA quality (size) and quantity were checked by electrophoresis in 0.8 % (w/v) horizontal agarose gel run in 0.5 % TBE (tris–borate-ethylenediaminete-triacetate, pH 8.3) buffer and stained with 0.9 µg mL^−1^ ethidium bromide (Sambrook et al. 1989). A molecular size marker (1-kb ladder) was used as the reference.

Dominant bacterial communities were distinguished by DGGE analysis and electrophoresis performed with a D-Code Universal Mutation Detection System (BioRad Laboratories, USA). A 1 µL aliquot (roughly 5–10 ng in undiluted form) of each DNA was amplified by PCR mixture containing: 5 µL of 10×·buffer (Sigma Aldrich Co.), 6.0 µL of 25 mM MgCl_2_ (Sigma Aldrich Co.), 1.2 µL of 20 mg ml^−1^ BSA (Sigma Aldrich Co.), 0.4 µL of 25 mM dNTP (Sigma Aldrich Co.), 0.5 µL of 20 µM in each primer, 0.2 µL of 5 U µL^−1^ Taq DNA polymerase (Sigma Aldrich Co.) and 35.2 µL of PCR grade water, in a total volume of 50 µL. The primers used were 341f with GC clamp (5′-GC-CC TAC GGG AGG CAG CAG-3′) complementary to position 341–357 and 907r (CCG TCA ATT CMT TTG AGT TT) complementary to positions 926–907 (*Escherichia coli* numbering) (Muyzer et al. 1993, 1998). The samples were loaded on 6 % acrylamide gel with a denaturing gradient of 35–70 % (where 100 % denaturant is 7 M urea and 40 % formamide). The gels were run at 60 V for 17 h at 60 °C. The electrophoretic products were stained by gently agitating the gel for 30 min in 100 mL of 1 × TAE containing 5 µL 1:10000 dilution of SYBR Gold nucleic acid stain (Invitrogen, Life Technologies, UK) in DMSO. DGGE banding patterns were visualized with UV transillumination and photographed using the Gel Doc 2000 gel documentation system (BioRad Laboratories, USA). DGGE gel images were analyzed by Quantity One software in the GelDoc gel documentation system (BioRad Laboratories, USA). Gel bands were identified using GelCompar software to create the presence–absence matrix described by Crump and Hobbie (2005). Each band represents a bacterial Operational Taxonomic Unit (OTU). The presence or absence of a band in each line was converted to binary matrix to access data for statistical analysis.

### Functional diversity of the microbial community

Filter concentrated ice and cryoconite suspensions have been adjusted to optical transmittance of 0.9. 100 µL aliquots of each suspension were added to each well of Ecoplate microplates (Biolog Inc., Hayward, CA, USA). The plates were incubated in darkness at 4 °C, the color development was measured at 590 nm with a microplate reader (OmniLog) and cellular respiration was measured kinetically by determining the colorimetric reduction of tetrazolium dye. Data were collected approximately twice a week over a 65-day period. The EcoPlate Biolog assays assessed the ability of a mixed microbial community to utilize any of 31 carbon compounds as the sole carbon source (+one control well with no carbon). Absorbance data from the different reading times (given in OmniLog arbitrary units) were first blanked against the time “zero” reading and then the values were blanked against the respective control well containing no carbon source. Positive values were scored as the communities ability to utilize given carbon source. The metabolic diversity of microbial communities was estimated as substrate richness (the number of substrates utilized). To compare the effect of a specific treatment, substrate utilization data were also subdivided into 5 substrate categories representing different substrate groups (carbohydrates, carboxylic and acetic acids, polymers, amino acids and amines) (Weber and Legge 2009).

### Statistics

Simple regression analysis between biological and environmental factors was carried out in STATISTICA v. 9 (StatSoft). A principal correspondence analysis was conducted using a statistical package—Canoco 4.5 for Windows v. (Ter Braak and Šmilauer 2002) and STATISTICA v. 9 (StatSoft).

## Results

### Environmental factors

Mean values of physico-chemical parameters of investigated surface ice samples are presented in Table 1; the complete data are presented in Table S2, Supplementary Materials. Surface ice total organic carbon (TOC) levels varied slightly in both glaciers with a higher mean value in Hans Glacier. At Hans Glacier, the amount ranged from 0.97 mg L^−1^ (HI3) to 5.45 mg L^−1^ (HI2), and in Werenskiold Glacier from 0.92 mg L^−1^ (WI3) to 2.90 mg L^−1^ (WI5). In majority of the samples, TOC was composed of dissolved carbon. Total nitrogen amounts measured in ice samples were at their highest in points nearest the snow line in both Hans and Werenskiold Glaciers (0.40 and 0.30 mg L^−1^, respectively). Organic nitrogen dominated in all samples. Inorganic nitrogen fraction was composed mainly of nitrates. Total phosphorus in Hans Glaciers ice samples exhibited highest values at glaciers terminus (HI1—0.249 mg L^−1^) and lowest at the snow line area (HI5—0.050 mg L^−1^). At Werenskiold Glacier, highest and lowest total phosphorus amounts were noted in neighboring areas (WI3—0.275 mg L^−1^; WI2—0.047 mg L^−1^). Organically bound P fraction dominated in high phosphorus level sites, while in low phosphorus sites, organic and inorganic P fraction contributed equally to total P contents. The C/N/P ratios for Hans and Werenskiold Glaciers surface ice were 25:2:1 and 14:1.4:1, respectively. Total chlorophyll concentrations on Werenskiold Glacier showed a rising trend with distance from glacier terminus (WI1—1.50 µg L^−1^; WI5—8.61 µg L^−1^) while on Hans Glacier no such trend could be observed with highest and lowest chlorophyll amounts registered at neighboring sites (HI4—7.11 µg L^−1^; HI3—1.59 µg L^−1^). Total seston reached highest values of 561.5 mg L^−1^ at Hans Glaciers lowest site (HI1), whereas on Werenskiold Glacier the highest site was most abundant in particulates (WI5—283.0 mg L^−1^). Seston was composed mostly of organic material. Noteworthy are the elevated SUVA measurements on Werenskiold Glacier, twice as high as on Hans Glacier surface. Mean cation (Na^+^, K^+^, Ca^2+^, Mg^2+^) and anion (Cl^−^, SO_4_^2 −^) amounts for Hans Glacier surface ice were as follows: 1.40 mg L^−1^, 0.27 mg L^−1^, 2.93 mg L^−1^, 0.27 mg L^−1^, 0.80 mg L^−1^ and 0.23 mg L^−1^, respectively. Analogous Werenskiold Glacier samples displayed following concentrations of the respective ions (1.22 mg L^−1^, 0.28 mg L^−1^, 3.46 mg L^−1^, 0.25 mg L^−1^, 0.49 mg L^−1^, 0.11 mg L^−1^).

**Table 1:** Differences in chlorophyll and pheophytin concentrations, particulates, pH, carbon (*TOC* total organic carbon; *DOC* dissolved organic carbon; *POC* particulate organic carbon), nitrogen, phosphorus and other mineral contents in samples used for microbiological analysis

Samples (mean values)	HI	WI	HC	WC
TOC (mg L^−1^)	2.78	2.00	20.96	8.06
DOC (mg L^−1^)	1.76	0.96	15.11	7.45
POC (mg L^−1^)	1.01	1.04	5.85	0.62
SUVA	9.02	18.64	2.33	2.80
pH	3.47	3.80	4.63	4.51
NH_4_–N (mg L^−1^)	0.00	0.00	0.08	0.01
NO_3_–N (mg L^−1^)	0.01	0.05	0.02	0.05
Total nitrogen (mg L^−1^)	0.23	0.20	3.70	1.17
Organic nitrogen (mg L^−1^)	0.21	0.14	3.59	1.09
PO_4_–P (mg L^−1^)	0.02	0.03	0.21	0.31
Total phosphorus (mg L^−1^)	0.11	0.14	N/A	N/A
Organic phosphorus (mg L^−1^)	0.08	0.11	N/A	N/A
Na^+^ (mg L^−1^)	1.40	1.22	2.97	1.99
K^+^ (mg L^−1^)	0.27	0.28	1.65	0.94
Ca^2+^ (mg L^−1^)	2.93	3.46	3.69	3.30
Mg^2+^ (mg L^−1^)	0.27	0.25	0.80	0.33
Cl^−^ (mg L^−1^)	0.80	0.49	1.53	1.22
SO_4_^2−^ (mg L^−1^)	0.23	0.11	0.71	0.74
Total iron (Fe) (mg L^−1^)	0.02	0.02	N/A	N/A
Chlorophyll *a* (µg L^−1^)	1.84	1.33	45.05	11.68
Pheophytin (µg L^−1^)	2.18	2.67	41.45	8.74
Total chlorophyll (µg L^−1^)	4.02	4.00	86.48	20.46
Total seston (mg dry wt. L^−1^)	200.94	152.18	N/A	N/A
Organic seston (mg dry wt L^−1^)	193.26	148.28	N/A	N/A
Chlorophyll *a*/Pheophytin	0.76	0.53	1.14	1.7

*HI* Hans Glacier ice samples (*n* = 5); *WI* Werenskiold Glacier ice samples (*n* = 5), *HC* Hans Glacier cryoconite samples (*n* = 4); *WC* Werenskiold Glacier cryoconite samples (*n* = 5), *N/A* not analyzed

Mean values of physico-chemical parameters of investigated cryoconite samples are presented in Table 1; the complete data are presented in Table S3, Supplementary Materials. Mean total organic carbon amounts were higher in cryoconite samples from Hans Glacier (42.11 mg L^−1^ (mean 20.96) vs. 12.51 mg L^−1^ (mean 8.06) on Werenskiold Glacier). Lowest TOC concentrations were noted in cryoconite holes nearest the snow line on both glaciers (HC5—10.77 mg L^−1^; WC5—4.95 mg L^−1^). It was mainly composed of the dissolved fraction (HC mean—15.11 mg L^−1^, WC mean—7.45 mg L^−1^). Total nitrogen amounts were much higher on Hans Glacier, reaching 7.57 mg L^−1^ (1.41 mg L^−1^ on Werenskiold Glacier) with the majority of it being bound to organic matter. Phosphates were found in concentration up to 0.36 mg L^−1^ on Hans Glacier (HC4) and 0.96 mg L^−1^ on Werenskiold Glacier (WC1). High fluctuations of total chlorophyll levels were found in cryoconite holes of Hans Glacier (20.3—247.0 µg L^−1^), less so on Werenskiold Glacier (14.5—28.6 µg L^−1^). Mean cation (Na^+^, K^+^, Ca^2+^, Mg^2+^) and anion (Cl^−^, SO_4_^2 −^) amounts for Hans Glacier cryoconite holes were as follows: 2.97, 1.65, 3.69, 0.80, 1.53 and 0.71 mg L^−1^, respectively. Analogous Werenskiold Glacier samples displayed following concentrations of the respective ions (1.99 mg L^−1^, 0.94 mg L^−1^, 3.30 mg L^−1^, 0.33 mg L^−1^, 1.22 mg L^−1^, 0.74 mg L^−1^). The majority of the mean nutrient concentrations were higher in Hans Glacier cryoconite holes, with the exception of phosphates, nitrates and SUVA measurements.

### Microbial abundance and diversity

Mean values of biological parameters of investigated surface ice samples are presented in Table 2; the complete data are presented in Table S4, Supplementary Materials. Total microbial counts (TC) were rather stable along the transect on Hans Glacier, exhibiting maximum values of 7.33 × 10^4^ mL^−1^ at point HI5, nearest the snow line. TC on Werenskiold Glacier displayed higher values, up to 29.2 × 10^5^ mL^−1^ in point WI5. Dividing cell count (DC) was approx. tenfold lower than the TC measurements. The DC/TC (%) ratio displayed lowest values in the middle of the ablation zone on both Glaciers (HI3—5.71; WI3—7.13). The ratio increased on Werenskiold Glacier towards the terminus. Photoautotrophic count (PHAC) displayed highest values at the verge of the snow line at both glaciers (2.79 × 10^3^ mL^−1^—HI5; 8.44 × 10^3^ mL^−1^—WI5) although the PHAC/TC ratio coincided with those numbers only on Hans Glacier, whereas on Werenskiold Glacier it was the highest at the glacier terminus (WI1). Cyanobacteria dominated the supraglacial photoautotrophic communities; however, photosynthetic Eukaryotes were more numerous at the lowest point of the Hans Glacier and the highest point of the Werenskiold Glacier transects. The banding patterns of DGGE revealed dominant bacterial taxa. Band numbers increased at the Hans Glaciers terminus (17 OTU’s at HI1) on Werenskiold Glacier showed highest values (15 OTU’s) at the middle point of the transect (WI3) and near the snow line (WI5). Few responses from the surface ice communities were obtained on the Biolog EcoPlates, with highest values at Hans Glaciers terminus and point WI4 at Werenskiold Glacier.

**Table 2:** Differences in microbiological parameters of glacier surface microbes

Samples (mean values)	HI	WI	HC	WC
TC	6.29^a^	10.31^a^	2.41^b^	2.56^b^
DC	0.65^a^	1.01^a^	0.39	0.22
DC/TC (%)	10.35	13.48	16.28	9.34
PHAC	0.10^a^	0.38^a^	0.09^b^	0.05^b^
PHAC/TC (%)	1.53	5.11	3.51	1.69
%Cyan	68.31	66.30	69.98	66.80
%Eucar	31.69	33.72	30.02	33.22
DGGE (OTU)	13.00	12.80	14.00	18.00
EcoPlate (number of positive responses)	2.20	2.40	19.00	15.40
CFU	0.37^a^	0.47^a^	0.06^b^	0.14^b^
CFU/TC (%)	5.92	6.07	2.86	4.71

*TC* total microbial count, *DC* dividing cell count, *CFU* colony forming units on R2A agar, *PHAC* photoautotrophic cell count, *%Cyan* percentage contribution of cyanobacterial cells to photoautotrophic count, *%Eucar* percentage contribution of eukaryotic cells to photoautotrophic count; *DGGE* taxonomical diversity of samples given in operational taxonomic units (OTU’s), *EcoPlate* functional diversity given in positive response numbers on Biolog Ecoplates. *HI* Hans Glacier ice samples (*n* = 5); *WI* Werenskiold Glacier ice samples (*n* = 5), *HC* Hans Glacier cryoconite samples (*n* = 4); *WC* Werenskiold Glacier cryoconite samples (*n* = 5)

^a^×10^4^ mL^−1^

^b^×10^8^ g dry weight^−1^

Mean values of biological parameters of investigated cryoconite samples are presented in Table 2; the complete data are presented in Table S5, Supplementary Materials. Total microbial counts in cryoconite sediment remained very stable along the Hans Glaciers ablation zone, with a maximum of 2.58 × 10^8^ g^−1^ d.w. TC on Werenskiold Glacier varied along the transect, displaying lowest values at both ends of the transect (WC1—0.74 × 10^8^ g^−1^ d.w.; WC5—0.82 × 10^8^ g^−1^ d.w.). The DC/TC ratio of the cryoconite samples at Hans Glacier was the highest near its terminus, on Werenskiold Glacier; however, this ratio showed maximal values at the snow line. Photoautotrophic to total cell count ratio has displayed a similar trend on both examined glaciers, with low values near the snow line (HC5—0.75 %; WC5—0.78 %) and highest closer to the terminus (HC2—7.39; WC2—2.53). Cyanobacteria dominated the photoautotrophic community in all cryoconite samples. Taxonomic diversity (OTU numbers) increased in samples from both Glaciers towards the terminus. The functional diversity (EcoPlate positive responses) showed a similar trend. CFU to TC ratio displayed a decreasing trend toward the terminus along Hans Glaciers ablation zone, whereas in Werenskiold Glaciers cryoconite holes, it was the highest in the middle of the transect.

**Fig. 2:**
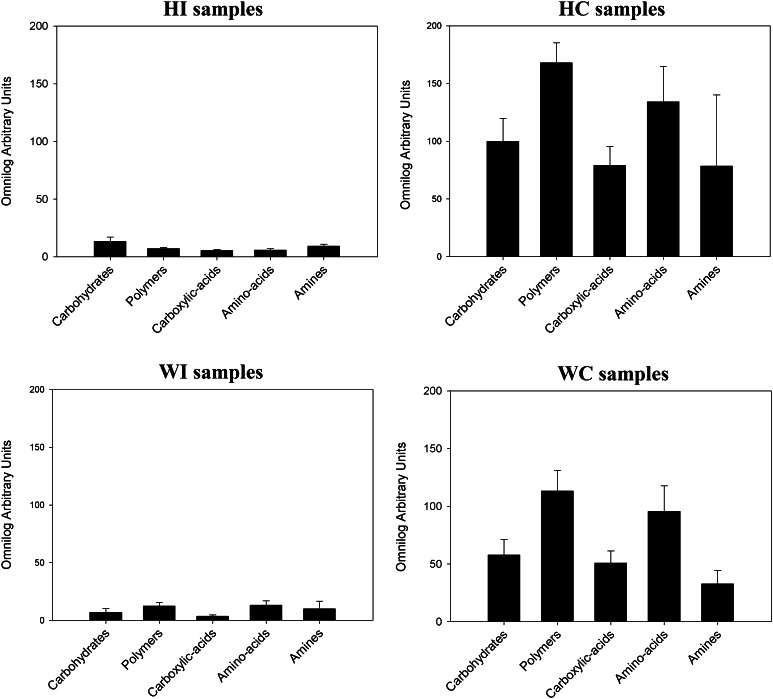
Well color development calculated from Omnilog Arbitrary Unit values of substrate utilization on Biolog EcoPlates by glacial microbial communities. Substrates were divided into five categories: carbohydrates (*n* = 10), polymers (*n* = 4), carboxylic and acetic acids (*n* = 9), amino acids (*n* = 6), amines/amides (*n* = 2). HI—Hans Glacier surface ice samples (*n* = 5), WI—Werenskiold Glacier surface ice samples (*n* = 5), HC—Hans Glacier cryoconite samples (*n* = 4), WC—Werenskiold Glacier cryoconite samples (*n* = 5)

Substrate utilizing abilities of the cryoconite communities were much broader than the ice surface ones in both glaciers (Fig. 2). Tetrazolium dye reduction rates were higher in cryoconites from Hans Glacier than that of Werenskiold Glacier. In all but one sample type (HI), the preferable carbon source was polymers. The second most frequently utilized compound group was amino acids. Pyruvic acid methyl ester, d-cellobiose, β-methyl-d-glucoside, α-d-lactose, l-asparagine were the most actively metabolized compounds by surface ice microbiota of Hans Glacier, whereas on Werenskiold Glacier pyruvic acid methyl ester, l-arginine, glycyl-l-glutamic acid, Tween 80 and Tween 40 were preferable. The compound utilization pattern was similar for cryoconite communities of both glaciers, with l-asparagine, Tween 80, l-arginine and α-cyclodextrin as popular carbon sources (data not shown).

### Statistics

Simple regression analysis was conducted between biological and physico-chemical parameters in surface ice and in cryoconite samples. Significant correlation (< 0.05) has been displayed in Table S6 (a–d), Supplementary Materials.

Basic microbial parameters of abundance (TC) and diversity (DGGE, EcoPlate) in Hans Glacier surface ice samples (HI), positively correlated with POC, eukaryotic photoautotroph contribution, pH and NH_4_^+^ levels. Correlations in Hans Glaciers cryoconite samples (HC) included a positive one between Mg^2+^ amounts and the dividing to total cell ratio (DC/TC) and photoautotroph contribution (PHAC/TC). The culturable to total cell ratio (CFU/TC) displayed negative correlations microbial abundance and diversity parameters. The anticipated correlations with distance from glacier edge included the PHAC/TC ratio (positive in surface ice, negative in cryoconite holes), TC (negative in cryoconite holes), the CFU/TC ratio (positive in cryoconite holes). In both the surface ice and the meltholes, the taxonomic diversity positively correlated with the functional diversity.

Higher numbers of statistically significant correlations emerged in Werenskiold Glacier samples. Microbial abundance in surface ice samples (WI) correlated positively with distance from glacier edge, organic nitrogen and chlorophyll *a* contents. The PHAC/TC and DC/TC ratios displayed similar negative correlations: with distance from glacier terminus, total and organic phosphorus contents, chlorophyll *a* and seston amounts. Taxonomic diversity positively correlated with ammonia and iron concentrations. The CFU/TC ratio displayed positive correlations with aromatic carbon contents (SUVA) but a negative with the whole dissolved fraction (DOC). In cryoconite hole samples (WC), negative correlations prevailed. Total and dissolved organic carbon fractions displayed negative correlations with distance from glacier terminus and the CFU/TC ratio. This ratio, like in surface ice samples, correlated positively with the SUVA measurements. Taxonomic diversity displayed a negative correlation with PO_4_^3−^ concentrations and TC with ammonia amounts.

**Fig. 3:**
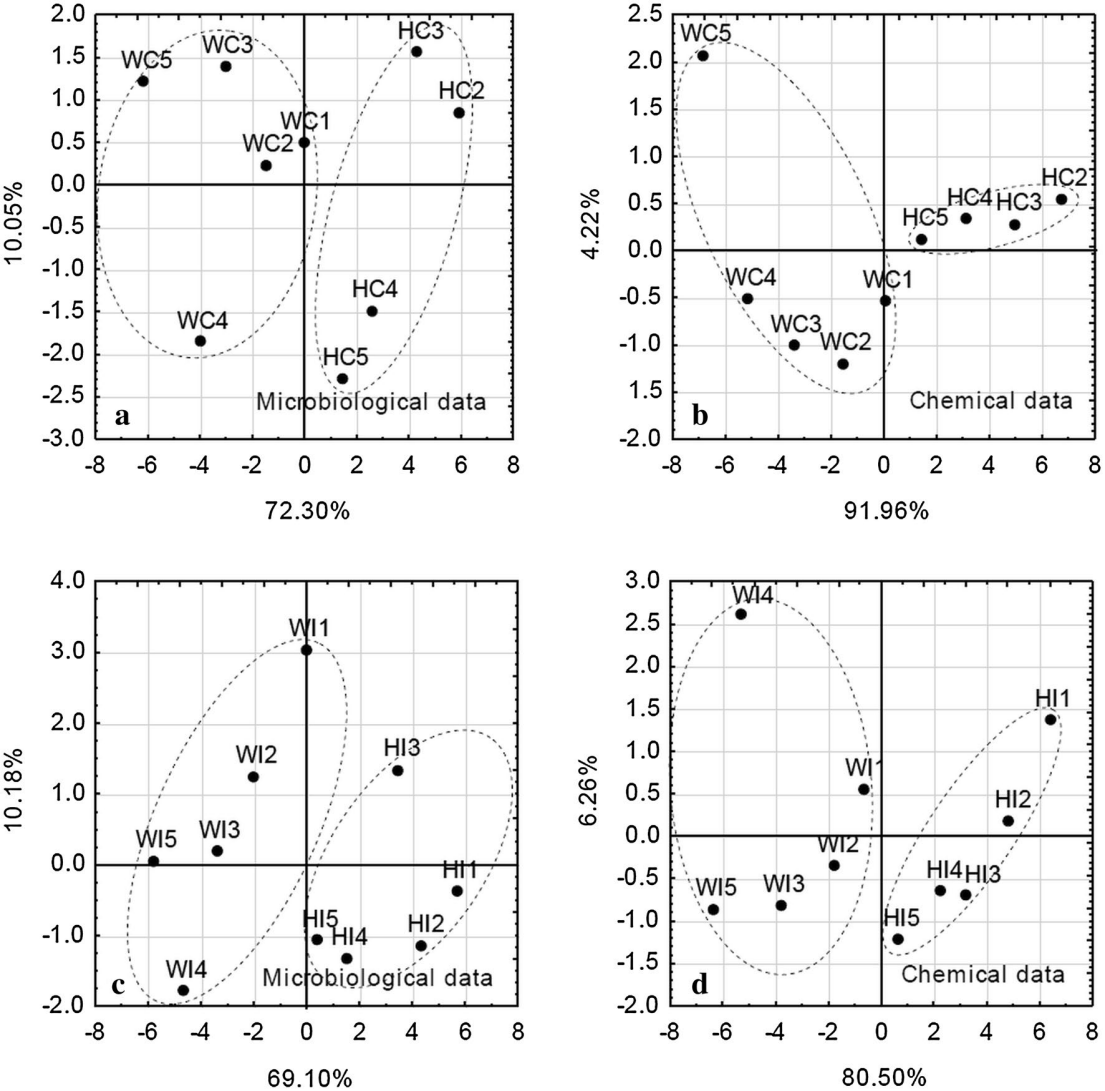
Principal component analysis clustering of sampling points based on physico-chemical (**b**, **d**) and microbiological data (**a**, **c**). WI—Werenskiold Glacier surface ice samples, HC—Hans Glacier cryoconite samples, WC—Werenskiold Glacier cryoconite samples

The Principal Component Analysis (PCA) of chemical and microbiological parameters shows a clear distinction between Hans and Werenskiold Glacier samples (Fig. 3). The clustering by chemical data shows the samples more homogenous within a group than the clustering by microbiological parameters. The spatial design of the sampling transect is reflected in the clustering of the samples, but in some cases single points diverge from the group—point WC4 in the microbiological clustering, sample WC5 and WI4 in chemical parameter clustering.

**Fig. 4:**
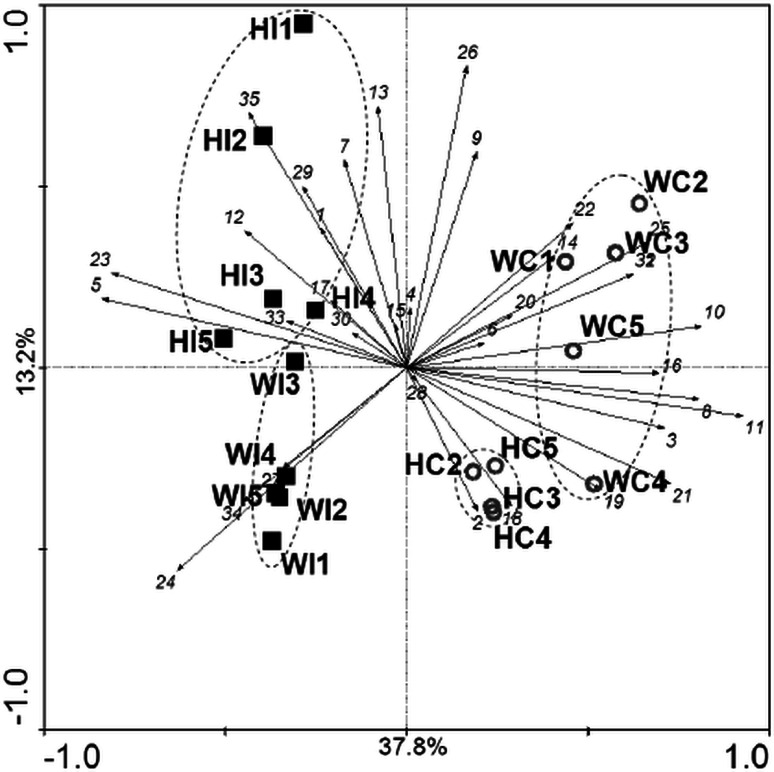
Principal component analysis of bacterial taxonomic structure (based on OTUs relations) in surface ice (*black squares*) and cryoconite samples (*circles*). HI—Hans Glacier surface ice samples, WI—Werenskiold Glacier surface ice samples, HC—Hans Glacier cryoconite samples, WC—Werenskiold Glacier cryoconite samples, 1–35—DGGE band numbers

The PCA of the dominant bacterial taxa structure based on the relations of OTU’s in the denaturating gel reveals that the bacteriocenoses in surface ice and cryoconite holes differ, but also shows differences between glaciers (Fig. 4). The surface ice samples from Werenskiold Glacier form a rather homogenous group, whereas Hans Glacier ice samples are more diverse. An opposite situation can be observed with cryoconite hole samples, with the Hans Glacier points forming a very tight cluster.

## Discussion

Microbial communities and their development on two Arctic glaciers were compared. Surface ice and cryoconite holes were examined in terms of chemical composition, microbial abundance and diversity. Gathered data served to describe supraglacial habitats on ablation zones of Hans and Werenskiold Glaciers and to compare microbe–environment interactions on those different type glaciers.

### Data comparison and nutrient sources

DOC levels in surface ice of Hans and Werenskiold Glaciers were in line with the findings of Zarsky et al. (2013) for Aldegonda Glacier and Stibal et al. (2008) for Werenskiold glacier. Both these authors attribute the present organic carbon to allochtonic inputs. Hans Glacier surface displayed elevated TOC, DOC and seston levels compared to Werenskiold Glacier, hinting that the source and intensity of allochtonic carbon supply may be different for each of the glaciers. Differences between the glaciers that were only slight in surface ice samples were more pronounced in cryoconite samples. Organic carbon, ammonia and organic nitrogen levels were much higher in Hans Glacier cryoconite holes. This suggests yet again a different nutrient source. The parameters of both glaciers were also much higher than those reported from McMurdo valley glaciers (Porazinska et al. 2004, Foreman et al. 2007) and previous studies on Werenskiold Glacier (Stibal et al. 2008). The elevated concentrations of some inorganic ions (Na^+^, K^+^, Mg^2+^, Ca^2+^) in Hans Glacier cryoconite samples may be attributable to marine aerosol influence (McInnes et al. 1996). The cause for such high nutrient levels may be found in the glacier-adjacent sites. Hans Glacier lies in the vicinity of little auk and several other birds’ breeding sites, rich tundra formations and it terminates in Hornsund fjord (Pulina et al. 2003). Tidewater glacier front areas have been recognized as important feeding sites for birds and sea mammals due to fresh-salt water mixing induced death of marine plankton species (Lydersen et al. 2014). Those sites could be important nutrient sources, as debris in the form of lichen and moss fragments as well as feathers and pellets have been observed by the authors on the ablation zone of Hans Glacier. The allochtonous organic matter source for Werenskiold glacier is not so easy to pinpoint. One suggestion could be the vast barren glacial forefield, where according to Bardget et al. (2007) ancient carbon can be traced. One of the characteristic of this carbon is its high aromatic content, which was predominantly detected (SUVA) on the surface of Werenskiold Glacier in our study.

Despite differences in some essential nutrient contents between the surface habitats of those glaciers, the microbial numbers are comparable, suggesting that other factors may control cell densities, like flushing (Stibal et al. 2008), viral-induced mortality (Bellas et al. 2013) or/and predatory ciliate grazing (Mieczan et al. 2013). However, some parameters indicate that nutrients could exert some changes in the trophic structure of supraglacial communities. According to Gasol and Duarte (2000), higher nutrient levels should shift the balance towards photoautotroph dominance on the more fertile Hans Glacier. Yet, the PHAC/TC ratio in surface ice was considerably higher in Werenskiold Glacier samples. Dodds and Cole (2007) point out that the proposed photoautotroph dominance scenario applies to high-autotrophy environments, whereas in low-autotrophy habitats with high allochtonic input, heterotroph dominance should be observed.

The dividing to total cell ratio (DC/TC) was higher in cryoconite holes of Hans Glacier, suggesting that the higher nutrient levels are beneficial for cell fission rates. However, the experiments of Säwstrom et al. (2007), involving enrichment of cryoconite microbiota in essential nutrients, did not produce a significant increase in cell doubling rates, which implies the influence of other factors, including higher cryoconite temperature or even different properties on the microbial community itself.

The PCA clustered the samples according to the bacterial taxonomic structure. It revealed that the bacteriocenoses cluster together within one sample type, which is consistent with the observations of Edwards et al. (2011, 2013). The most coherent groups were formed by the Hans Glacier cryoconite samples and Werenskiold Glacier surface ice samples. Taxonomically, similar bacterial communities in different locations are thought to be the effect of strong selective factors (Cameron et al. 2012). Given that these habitats represent the highest (HC) and the lowest (WI) nutrient levels presented in this study, we assume that this could be one of the responsible factors. In comparison to surface ice, cryoconite holes were described as microbial refugia on glacier surfaces, protecting the cells from UV radiation, flush-out, freezing and other factors. In this respect, harsh physical conditions and low nutrient availability could exert enough selective pressure to unify the bacterial community across the ablation zone, whereas in cryoconite holes of Hans Glacier biotic interactions and competition could do the same.

### Supraglacial community development

Microbial community development in this study involves mainly the response of the supraglacial microbes to the passing of the snow line during the ablation period and exposure of the ice to allochtonic influences.

Nutrient and microbial cell supply to the surface ice by the melting snow cover has been observed previously (Amato et al. 2007; Telling et al. 2011). As the passing of the snow line is a seasonally progressing phenomenon, one can conclude that the entire ablation zone had to be submitted to snow-derived nutritional and microbial augmentation, with sites near terminus being the first to experience this event. The gradual reaction of the microbial community should, therefore, be observed towards the glacier terminus. The immediate increase in microbial quantity was only observed in the Werenskiold Glacier surface ice samples (WI5), whereas in cryoconite samples of this glacier the numbers rose in point WI4, indicating a delayed response. The snowmelt had seemingly no effect on the Hans Glacier supraglacial microbocenosis. However, chlorophyll levels in cryoconite holes from point 4 were suprisingly high, with higher PHAC/TC ratio. This could suggest that although the community as a whole seems unaffected, the photoautotrophs proliferate, producing large quantities of photosynthetic pigments. This is consistent, with the findings of Persson et al. (2010), who state that photoautotrophs are more flexible than heterotrophs in response to fluctuations in nutrient levels.

The PHAC/TC ratio remains a curious issue. In Hans Glacier surface ice, this ratio declined towards the terminus, which is consistent with the model proposed by Stibal et al. 2012, where the glacial margin acts as CO_2_ source, turning towards net-autotrophy towards snow accumulation area. However, in cryoconite holes of Werenskiold Glacier, the opposite could be observed. Furthermore, the chlorophyll *a* to pheophytin ratio, as an indicator of “health” condition of photoautotrophs (Camacho and de Wit 2003), also increased in Werenskiold Glacier cryoconites towards the terminus. An explanation can be given again by Dodds and Cole (2007), if we consider the cryoconite holes as high-autotrophy habitats with high allochtonic input. This scenario implies an increase of photoautotrophic activity and abundance.

Siegler and Zeyer (2004) proposed the CFU to total cell count ratio to be the opportunist part of the community. The ratio is higher in newly established or frequently disturbed habitats (Zdanowski et al. 2013). In this regard, the decline of this ratio in cryoconite samples of both examined glaciers towards terminus hits that those communities develop over time, forming complex trophic interactions, that cannot be imitated by the agar medium.

### Influencing factors

Very few correlations have emerged between microbial abundance and other factors. A recurring phenomenon was positive correlations between TC and several parameters involving photoautotrophs like the PHAC/TC ratio, chlorophyll *a* content and Eukaryotic or Cyanobacterial autotroph percentage. This suggests that the microbial community depends not solemnly on allochtonic nutrients, but also benefits from in situ primary production. Investigations of Stibal et al. (2007) on Werenskiold Glacier involving inorganic carbon uptake in comparison to total carbon amounts in cryoconite holes hint that microbial photosynthesis is of negligible importance to the supraglacial habitat. However, analysis of cryoconite organic matter revealed it to be in large part recalcitrant and low availability compounds like long chain n-alkanes and wax esters (Xu et al. 2010). In those conditions, excretion of low molecular organic molecules like free amino acids by photoautotrophs (Myklestad 1995) could greatly benefit the microbial community.

Photoautotrops, especially eukaryotic algae, have in surface samples displayed positive correlations with nitrogen levels, pointing to nitrogen limitation in this habitat for non-nitrogen-fixing primary producers. The C/N/P ratios that are comparable to the ratios published by Stibal et al. 2008 for Werenskiold Glacier at the end of ablation season also point towards nitrogen limitation. Cyanobacteria (presumable nitrogen fixers) could benefit the microbial community in such conditions, yet no positive correlations emerged, hinting that nitrogen fixation might be impaired by an undisclosed factor. In the cryoconite holes, however, cyanobaterial presence greatly improves microbial numbers and diversity, suggesting better conditions for this process. The heightened nutrient content may be the cause, but also the lower UV radiation intensity (absorbed by the water layer) which was postulated by Solheim et al. (2002) to impair nitrogen fixation. High microbial numbers in cryoconite hole sediments imply high nitrogen demand and in consequence rapid depletion of the snow-derived N-compounds. Therefore, cyanobacterially fixed nitrogen may play a crucial role in community development. Telling et al. (2011) demonstrated that nitrogen fixation may cover microbial nitrogen demands when allochtonic sources are scarce.

An interesting issue emerged in Werenskiold Glacier surface ice samples. The photoautotroph part of the community responded negatively to heightened phosphorus, iron and seston contents, hinting that a delicate balance exists in this habitat. The PHAC/TC ratio shifts towards the heterotrophs in response to allochtonic nutrient supplementation, proving yet again, that according to Dodds and Cole (2007), Werenskiold Glacier surface ice can be seen as a low-autotrophy habitat.

The CFU/TC ratio as an opportunist contribution indicator displayed several negative correlations with seemingly growth essential nutrient concentrations, especially DOC, but also with microbial abundance and diversity parameters, proving it to be the indicator of stressful conditions for microbes. It displayed positive correlation with the aromaticity content of DOC. Aromatic compounds are known as a last resort carbon supply, when other sources are depleted, because of their complex biodegradation process and, therefore, low availability to microbial consumers (Kanaly and Harayama 2000).

## Conclusions

The presented data let us conclude that different type glaciers (Hans and Werenskiold) originating from the same ice cap due differ, especially in nutrient content, but also in supraglacial microbial community structure. The reason for those discrepancies may be sought in allochtonic inputs of wind and bird delivered materials from adjacent environments. Hans Glacier appeared to be under more intense allochtonic enrichment than Werenskiold Glacier, probably due to the nature of its surroundings. Although this phenomenon seemed to affect the microbial numbers only moderately, it influenced the quality of the microbial community, including not only the taxonomic structure, but also the balance between heterotrophic and photoautotrophic cell distribution. The communities development after snow cover retreat showed that the allochtonic enrichment on Hans Glacier may overwhelm the effect of the melting snow and corrupt the development of the microbial community observed on Werenskiold Glacier. Nitrogen emerged as one of the few candidates to affect microbial cell densities and diversity, especially in surface ice, where stressful condition might have impaired important biochemical processes like nitrogen fixation.

## Electronic supplementary material

Supplementary material 1 (DOCX 38 kb)
