# Supplementary material for: Microbial community development on the surface of Hans and Werenskiold Glaciers (Svalbard, Arctic): a comparison
Source: Extremophiles. 2015 Jun 24;19(5):885–97. doi: 10.1007/s00792-015-0764-z (PMC4546695; doi:10.1007/s00792-015-0764-z)
Supplement: Supplementary file 1 — Supplementary material 1 (DOCX 38 kb) [file 792_2015_764_MOESM1_ESM.docx]

Table S1. Locations of the sampling points.

| **Sampling points** | **HI1** | **HI2/HC2** | **HI3/HC3** | **HI4/HC4** | **HI5/HC5** | **WI1/WC1** | **WI2/WC2** | **WI3/WC3** | **WI4/WC4** | **WI5/WC5** |
| --- | --- | --- | --- | --- | --- | --- | --- | --- | --- | --- |
| **Longitude (E)** | 15°35'55.0 | 15°39'27.8 | 15°39'11.3 | 15°38'10.9 | 15°37'54.9 | 15°15'33.4 | 15°17'23.3 | 15°19'29.7 | 77°04'14.3 | 15°23'30.5 |
| **Latitude (N)** | 77°01'04.2 | 77°01'33.2 | 77°02'10.9 | 77°02'48.6 | 77°03'47.6 | 77°04'34.4 | 77°04'32.8 | 77°04'24.5 | 15°21'22.7 | 77°04'05.4 |

Table S2. Differences in chlorophyll and pheophytin concentrations, particulates, pH, carbon (TOC – total organic carbon; DOC – dissolved organic carbon; POC – particulate organic carbon), nitrogen, phosphorus and other mineral contents in samples used for microbiological analysis. HI1-HI5 – Hans Glacier ice samples; WI1-WI5 – Werenskiold Glacier ice samples.

| **Sampling points** | **HI1** | **HI2** | **HI3** | **HI4** | **HI5** | **Mean** | **WI1** | **WI2** | **WI3** | **WI4** | **WI5** | **Mean** |
| --- | --- | --- | --- | --- | --- | --- | --- | --- | --- | --- | --- | --- |
| **TOC** (mg L^-1^) | 2.562 | 5.452 | 0.971 | 1.476 | 3.417 | **2.78** | 2.819 | 1.383 | 0.927 | 1.991 | 2.903 | **2.00** |
| **DOC** (mg L^-1^) | 1.666 | 4.099 | 0.654 | 1.063 | 1.329 | **1.76** | 1.055 | 0.829 | 0.534 | 0.528 | 1.867 | **0.96** |
| **POC** (mg L^-1^) | 0.896 | 1.353 | 0.317 | 0.413 | 2.088 | **1.01** | 1.764 | 0.554 | 0.393 | 1.463 | 1.036 | **1.04** |
| **SUVA** | 8.70 | 3.22 | 17.13 | 6.40 | 9.63 | **9.02** | 19.72 | 16.77 | 31.27 | 17.99 | 7.45 | **18.64** |
| **pH** | 3.61 | 3.48 | 3.43 | 3.34 | 3.51 | **3.47** | 4.21 | 3.86 | 3.39 | 3.82 | 3.70 | **3.80** |
| **NH_4_-N** (mg L^-1^) | 0.002 | 0.000 | 0.001 | 0.000 | 0.001 | **0.00** | 0.000 | 0.000 | 0.001 | 0.000 | 0.000 | **0.00** |
| **NO_3_-N** (mg L^-1^) | 0.009 | 0.010 | 0.003 | 0.024 | 0.027 | **0.01** | 0.118 | 0.027 | 0.017 | 0.087 | 0.013 | **0.05** |
| **Total nitrogen** (mg L^-1^) | 0.317 | 0.140 | 0.106 | 0.182 | 0.404 | **0.23** | 0.282 | 0.106 | 0.109 | 0.184 | 0.304 | **0.20** |
| **Organic nitrogen** (mg L^-1^) | 0.305 | 0.128 | 0.098 | 0.132 | 0.376 | **0.21** | 0.162 | 0.076 | 0.084 | 0.082 | 0.284 | **0.14** |
| **PO_4_-P** (mg L^-1^) | 0.022 | 0.019 | 0.022 | 0.021 | 0.028 | **0.02** | 0.025 | 0.021 | 0.038 | 0.024 | 0.024 | **0.03** |
| **Total phosphorus** (mg L^-1^) | 0.249 | 0.074 | 0.053 | 0.104 | 0.050 | **0.11** | 0.065 | 0.047 | 0.275 | 0.122 | 0.168 | **0.14** |
| **Organic phosphorus** (mg L^-1^) | 0.227 | 0.054 | 0.031 | 0.082 | 0.023 | **0.08** | 0.040 | 0.026 | 0.237 | 0.098 | 0.144 | **0.11** |
| **Na^+^** (mg L^-1^) | 1.32 | 1.29 | 1.18 | 1.32 | 1.91 | **1.40** | 1.28 | 0.97 | 1.06 | 0.96 | 1.83 | **1.22** |
| **K^+^** (mg L^-1^) | 0.25 | 0.34 | 0.21 | 0.25 | 0.30 | **0.27** | 0.31 | 0.19 | 0.27 | 0.15 | 0.50 | **0.28** |
| **Ca^2+^** (mg L^-1^) | 2.00 | 2.61 | 4.06 | 3.39 | 2.60 | **2.93** | 3.69 | 2.51 | 4.11 | 3.75 | 3.22 | **3.46** |
| **Mg^2+^** (mg L^-1^) | 0.35 | 0.20 | 0.26 | 0.19 | 0.37 | **0.27** | 0.27 | 0.17 | 0.27 | 0.23 | 0.32 | **0.25** |
| **Cl^-^** (mg L^-1^) | 0.42 | 0.69 | 0.24 | 0.95 | 1.71 | **0.80** | 0.75 | 0.55 | 0.31 | 0.25 | 0.60 | **0.49** |
| **SO_4_^2 -^**(mg L^-1^) | 0.37 | 0.18 | 0.11 | 0.11 | 0.40 | **0.23** | 0.22 | 0.04 | 0.10 | 0.12 | 0.07 | **0.11** |
| **Total iron (Fe)** (mg L^-1^) | 0.029 | 0.017 | 0.022 | 0.022 | 0.020 | **0.02** | 0.013 | 0.019 | 0.033 | 0.020 | 0.023 | **0.02** |
| **Chlorophyll *a*** (µg L^-1^) | 1.74 | 0.53 | 0.53 | 3.61 | 2.81 | **1.84** | 0.27 | 0.67 | 1.43 | 1.60 | 2.67 | **1.33** |
| **Pheophytin** (µg L^-1^) | 2.29 | 1.62 | 1.06 | 3.50 | 2.43 | **2.18** | 1.23 | 1.30 | 1.44 | 3.45 | 5.93 | **2.67** |
| **Total chlorophyll** (µg L^-1^) | 4.02 | 2.15 | 1.59 | 7.11 | 5.24 | **4.02** | 1.50 | 1.96 | 2.87 | 5.05 | 8.61 | **4.00** |
| **Total seston** (mg dry wt. L^-1^) | 561.5 | 82.3 | 97.8 | 166.8 | 96.3 | **200.94** | 49.3 | 38.3 | 213.3 | 177.0 | 283.0 | **152.18** |
| **Organic seston** (mg dry wt L^-1^) | 551.1 | 78.4 | 92.5 | 154.5 | 89.8 | **193.26** | 46.6 | 36.1 | 207.0 | 173.3 | 278.4 | **148.28** |
| **Chlorophyll *a*/ Pheophytin** | 0.76 | 0.33 | 0.50 | 1.03 | 1.16 | **0.76** | 0.22 | 0.52 | 0.99 | 0.46 | 0.45 | **0.53** |

Table S3. Differences in chlorophyll and pheophytin concentrations, particulates, pH, carbon (TOC – total organic carbon; DOC – dissolved organic carbon; POC – particulate organic carbon), nitrogen, phosphorus and other mineral contents in samples used for microbiological analysis. HC2-HC5 – Hans Glacier cryoconite samples; WC1-WC5 – Werenskiold Glacier cryoconite samples.

| **Sampling points** | **HC2** | **HC3** | **HC4** | **HC5** | **Mean** | **WC1** | **WC2** | **WC3** | **WC4** | **WC5** | **Mean** |
| --- | --- | --- | --- | --- | --- | --- | --- | --- | --- | --- | --- |
| **TOC** (mg L^-1^) | 11.680 | 19.290 | 42.110 | 10.770 | **20.96** | 12.510 | 8.720 | 6.432 | 7.696 | 4.956 | **8.06** |
| **DOC** (mg L^-1^) | 8.742 | 16.600 | 27.820 | 7.275 | **15.11** | 11.710 | 8.216 | 5.943 | 6.673 | 4.697 | **7.45** |
| **POC** (mg L^-1^) | 2.938 | 2.690 | 14.290 | 3.495 | **5.85** | 0.800 | 0.504 | 0.489 | 1.023 | 0.259 | **0.62** |
| **SUVA** | 3.24 | 1.74 | 1.52 | 2.80 | **2.33** | 1.96 | 1.73 | 4.04 | 3.21 | 3.04 | **2.80** |
| **pH** | 4.77 | 4.53 | 4.66 | 4.57 | **4.63** | 4.63 | 4.70 | 4.48 | 4.36 | 4.39 | **4.51** |
| **NH_4_-N** (mg L^-1^) | 0.033 | 0.084 | 0.174 | 0.040 | **0.08** | 0.012 | 0.006 | 0.002 | 0.004 | 0.008 | **0.01** |
| **NO_3_-N** (mg L^-1^) | 0.018 | 0.038 | 0.016 | 0.004 | **0.02** | 0.113 | 0.012 | 0.035 | 0.078 | 0.000 | **0.05** |
| **Total nitrogen** (mg L^-1^) | 1.453 | 3.399 | 7.576 | 2.358 | **3.70** | 1.058 | 1.268 | 0.960 | 1.411 | 1.151 | **1.17** |
| **Organic nitrogen** (mg L^-1^) | 1.399 | 3.275 | 7.386 | 2.312 | **3.59** | 0.933 | 1.250 | 0.923 | 1.317 | 1.002 | **1.09** |
| **PO_4_-P** (mg L^-1^) | 0.16 | 0.175 | 0.36 | 0.15 | **0.21** | 0.955 | 0.09 | 0.14 | 0.08 | 0.265 | **0.31** |
| **Na^+^** (mg L^-1^) | 2.18 | 2.41 | 4.26 | 3.03 | **2.97** | 2.14 | 1.64 | 1.39 | 2.35 | 2.43 | **1.99** |
| **K^+^** (mg L^-1^) | 1.15 | 1.54 | 2.30 | 1.60 | **1.65** | 1.15 | 1.03 | 0.59 | 0.94 | 1.01 | **0.94** |
| **Ca^2+^** (mg L^-1^) | 4.39 | 2.91 | 4.10 | 3.35 | **3.69** | 2.70 | 2.69 | 1.88 | 4.86 | 4.35 | **3.30** |
| **Mg^2+^** (mg L^-1^) | 1.25 | 0.73 | 0.76 | 0.46 | **0.80** | 0.38 | 0.36 | 0.20 | 0.40 | 0.32 | **0.33** |
| **Cl^-^** (mg L^-1^) | 1.12 | 1.21 | 2.35 | 1.45 | **1.53** | 1.01 | 0.82 | 0.84 | 1.84 | 1.57 | **1.22** |
| **SO_4_^2-^** (mg L^-1^) | 0.78 | 0.49 | 0.87 | 0.68 | **0.71** | 0.50 | 0.42 | 0.41 | 0.32 | 2.04 | **0.74** |
| **Chlorophyll  *a*** (µg L^-1^) | 9.2 | 18.7 | 125.6 | 26.7 | **45.05** | 19.6 | 10.8 | 20.0 | 2.7 | 5.3 | **11.68** |
| **Pheophytin** (µg L^-1^) | 11.2 | 14.5 | 121.4 | 18.7 | **41.45** | 5.3 | 6.9 | 8.5 | 11.8 | 11.2 | **8.74** |
| **Total chlorophyll** (µg L^-1^) | 20.3 | 33.2 | 247.0 | 45.4 | **86.48** | 24.9 | 17.7 | 28.6 | 14.5 | 16.6 | **20.46** |
| **Chlorophyll *a*/ Pheophytin** | 0.82 | 1.29 | 1.03 | 1.43 | **1.14** | 3.7 | 1.57 | 2.53 | 0.23 | 0.47 | **1.7** |

Table S4**.** Differences in microbiological parameters of surface ice microbes. TC – total microbial count, DC – dividing cell count, CFU – colony forming units on R2A agar, PHAC – photoautotrophic cell count, %Cyan – percentage contribution of cyanobacterial cells to photoautotrophic count, %Eucar - percentage contribution of eukaryotic cells to photoautotrophic count; DGGE – taxonomical diversity of samples given in operational taxonomic units (OTU’s), EcoPlate – functional diversity given in positive response numbers on Biolog Ecoplates. HI1-HI5 – Hans Glacier ice samples; WI1-WI5 – Werenskiold Glacier ice samples.

| **Sampling points** | **HI1** | **HI2** | **HI3** | **HI4** | **HI5** | **Mean** | **WI1** | **WI2** | **WI3** | **WI4** | **WI5** | **Mean** |
| --- | --- | --- | --- | --- | --- | --- | --- | --- | --- | --- | --- | --- |
| **TC** (x 10^4^ mL^-1^) | 5.63±0.47 | 6.88±0.67 | 5.90±0.49 | 5.70±0.57 | 7.33±0.28 | **6.29** | 1.99±0.18 | 3.61±0.14 | 9.45±0.83 | 7.30±0.52 | 29.2±1.22 | **10.31** |
| **DC** (x 10^3^ mL^-1^) | 5.32±0.91 | 8.91±1.36 | 3.37±0.83 | 7.82±0.63 | 7.28±0.92 | **6.54** | 4.45±1.21 | 7.06±1.78 | 6.74±1.52 | 7.06±0.62 | 25.2±2.65 | **10.10** |
| **DC/TC** (%) | 9.46 | 12.94 | 5.71 | 13.71 | 9.93 | **10.35** | 22.40 | 19.58 | 7.13 | 9.67 | 8.64 | **13.48** |
| **PHAC** (x10^3^ mL^-1^) | 0.20 | 0.11 | 0.21 | 1.69 | 2.79 | **1.00** | 1.53 | 2.43 | 2.96 | 3.70 | 8.44 | **3.81** |
| **PHAC/TC** (%) | 0.37 | 0.15 | 0.35 | 2.97 | 3.81 | **1.53** | 7.71 | 6.75 | 3.13 | 5.07 | 2.89 | **5.11** |
| **%Cyan** | 25.00 | 100.00 | 80.00 | 75.00 | 61.54 | **68.31** | 64.0 | 58.8 | 93.8 | 76.7 | 38.2 | **66.30** |
| **%Eucar** | 75.00 | 0.00 | 20.00 | 25.00 | 38.46 | **31.69** | 36.0 | 41.2 | 6.3 | 23.3 | 61.8 | **33.72** |
| **DGGE** (OTU) | 17 | 12 | 13 | 10 | 13 | **13.00** | 11 | 12 | 15 | 11 | 15 | **12.80** |
| **EcoPlate** (number of positive responses) | 5 | 1 | 1 | 1 | 3 | **2.20** | 0 | 2 | 2 | 5 | 3 | **2.40** |
| **CFU** (x 10^3^ mL^-1^) | 2.29 | 4.62 | 1.96 | 4.94 | 5.01 | **3.76** | 1.48 | 1.80 | 7.72 | 5.33 | 7.20 | **4.71** |
| **CFU/TC** (%) | 4.07 | 6.71 | 3.32 | 8.66 | 6.83 | **5.92** | 7.44 | 4.99 | 8.17 | 7.30 | 2.47 | **6.07** |

Table S5**.** Differences in microbiological parameters of cryoconite microbes. TC – total microbial count, DC – dividing cell count, CFU – colony forming units on R2A agar, PHAC – photoautotrophic cell count, %Cyan – percentage contribution of cyanobacterial cells to photoautotrophic count, %Eucar - percentage contribution of eukaryotic cells to photoautotrophic count; DGGE – taxonomical diversity of samples given in operational taxonomic units (OTU’s), EcoPlate – functional diversity given in positive response numbers on Biolog Ecoplates. HC2-HC5 – Hans Glacier cryoconite samples; WC1-WC5 – Werenskiold Glacier cryoconite samples.

| **Sampling points** | **HC2** | **HC3** | **HC4** | **HC5** | **Mean** | **WC1** | **WC2** | **WC3** | **WC4** | **WC5** | **Mean** |
| --- | --- | --- | --- | --- | --- | --- | --- | --- | --- | --- | --- |
| **TC** (x 10^8^ g d.w. ^-1^) | 2.58±0.11 | 2.46±0.16 | 2.40±0.29 | 2.20±0.29 | **2.41** | 0.74±0.09 | 3.39±0.46 | 3.98±0.41 | 3.87±0.30 | 0.82±0.44 | **2.56** |
| **DC** (x 10^7^ g d.w. ^-1^) | 5.08±0.71 | 3.72±0.24 | 4.17±0.67 | 2.86±0.71 | **3.96** | 0.60±0.13 | 0.71±0.16 | 4.81±0.45 | 4.22±0.18 | 1.11±0.11 | **2.29** |
| **DC/TC** (%) | 19.65 | 15.11 | 17.36 | 13.00 | **16.28** | 8.06 | 2.10 | 12.10 | 10.91 | 13.51 | **9.34** |
| **PHAC** (x 10^6^ g d.w. ^-1^) | 19.1 | 8.43 | 5.95 | 1.66 | **8.79** | 1.22 | 8.57 | 8.17 | 5.64 | 0.63 | **4.85** |
| **PHAC/TC** (%) | 7.39 | 3.42 | 2.48 | 0.75 | **3.51** | 1.64 | 2.53 | 2.06 | 1.46 | 0.78 | **1.69** |
| **%Cyan** | 88.73 | 73.68 | 56.41 | 61.11 | **69.98** | 61.8 | 68.8 | 72.4 | 69.5 | 61.5 | **66.80** |
| **%Eucar** | 11.27 | 26.32 | 43.59 | 38.89 | **30.02** | 38.2 | 31.3 | 27.6 | 30.5 | 38.5 | **33.22** |
| **DGGE** (OTU) | 15 | 16 | 13 | 12 | **14.00** | 19 | 19 | 19 | 16 | 17 | **18.00** |
| **EcoPlate** (number of positive responses) | 25 | 24 | 14 | 13 | **19.00** | 20 | 17 | 17 | 18 | 5 | **15.40** |
| **CFU** (x 10^6^ g d.w ^-1^) | 1.42 | 1.42 | 8.40 | 15.0 | **6.56** | 0.43 | 14.3 | 33.7 | 17.9 | 4.63 | **14.19** |
| **CFU/TC** (%) | 0.55 | 0.58 | 3.50 | 6.82 | **2.86** | 0.58 | 4.22 | 8.48 | 4.63 | 5.64 | **4.71** |

Table S6a. Correlation coefficients (*p<0.05, **p<0.01) between Hans Glacier surface ice chosen biological parameters and non-biological factors. TC – total microbial count, DC/TC – dividing to total cell count ratio, PHAC/TC – photoautotrophic cell count to total microbial count ratio, DGGE – taxonomical diversity of samples by taxonomic operational units (OTU’s), DISTANCE – distance from glacier edge, POC – particulate organic carbon, %Eucar - percentage contribution of eukaryotic cells to photoautotrophic count EcoPlate – functional diversity of samples by positive response numbers on Biolog EcoPlates), SUVA – aromaticity content of dissolved organic carbon.

| **HI samples** | TC | DC/TC | PHAC/TC | %Eucar | DGGE | EcoPlate |
| --- | --- | --- | --- | --- | --- | --- |
| %Eucar | -0.372 | -0.259 | 0.068 | 1.000 | 0.745 | 0.937** |
| SUVA | -0.265 | -0.934** | -0.100 | 0.169 | 0.220 | 0.000 |
| pH | 0.152 | -0.290 | -0.327 | 0.662 | 0.958** | 0.864* |
| NH_4_-N | -0.144 | -0.436 | -0.072 | 0.933** | 0.934** | 0.967** |
| NO_3_-N | 0.402 | 0.558 | 0.952** | 0.071 | -0.208 | 0.054 |
| Total iron | -0.689 | -0.304 | -0.161 | 0.925** | 0.570 | 0.781 |
| DGGE | 0.117 | -0.491 | -0.206 | 0.745 | 1.000 | 0.887* |
| DISTANCE | 0.588 | 0.098 | 0.855* | -0.338 | -0.341 | -0.366 |
| POC | 0.900* | 0.187 | 0.412 | 0.044 | 0.427 | 0.322 |

Table S6b**.** Correlation coefficients (*p<0.05, **p<0.01) between Hans Glacier cryoconite holes chosen biological parameters and non-biological factors. TC – total microbial count, DC/TC – dividing to total cell count ratio, PHAC/TC – photoautotrophic cell count to total microbial count ratio, DGGE – taxonomical diversity of samples by taxonomic operational units (OTU’s), DISTANCE – distance from glacier edge, CFU/TC– colony forming units on R2A agar to total microbial count ratio, %Cyan - percentage contribution of cyanobacterial cells to photoautotrophic count, EcoPlate – functional diversity of samples by positive response numbers on Biolog EcoPlates.

| **HC samples** | TC | DC/TC | PHAC/TC | %Cyan | DGGE | EcoPlate | CFU/TC |
| --- | --- | --- | --- | --- | --- | --- | --- |
| TC | 1.000 | 0.875 | 0.929* | 0.779 | 0.885* | 0.848 | -0.947* |
| PHAC/TC | 0.929* | 0.872 | 1.000 | 0.908* | 0.813 | 0.828 | -0.809 |
| %Cyan | 0.779 | 0.589 | 0.908* | 1.000 | 0.852 | 0.911* | -0.739 |
| DGGE | 0.885* | 0.558 | 0.813 | 0.852 | 1.000 | 0.989** | -0.963** |
| EcoPlate | 0.848 | 0.523 | 0.828 | 0.911* | 0.989** | 1.000 | -0.916* |
| Mg^2+^ | 0.927* | 0.947* | 0.982** | 0.816 | 0.726 | 0.726 | -0.767 |
| DISTANCE | -0.993** | -0.814 | -0.900* | -0.779 | -0.926* | -0.886* | 0.978** |

Table S6c. Correlation coefficients (*p<0.05, **p<0.01) between Werenskiold Glacier surface ice chosen biological parameters and non-biological factors. TC – total microbial count, DC/TC – dividing to total cell count ratio, PHAC/TC – photoautotrophic cell count to total microbial count ratio, DGGE – taxonomical diversity of samples by taxonomic operational units (OTU’s), DISTANCE – distance from glacier edge, CFU/TC– colony forming units on R2A agar to total microbial count ratio, DOC – dissolved organic carbon, %Eucar - percentage contribution of eukaryotic cells to photoautotrophic count, SUVA – aromaticity content of dissolved organic carbon.

| **WI samples** | TC | PHAC/TC | CFU/TC | %Eucar | DGGE |
| --- | --- | --- | --- | --- | --- |
| TC | 1.000 | -0.776 | -0.757 | 0.811* | 0.579 |
| DC/TC | -0.619 | 0.949** | 0.078 | -0.092 | -0.612 |
| CFU/TC | -0.757 | 0.280 | 1.000 | -0.877* | -0.287 |
| %Eucar | 0.811* | -0.264 | -0.877* | 1.000 | 0.105 |
| DOC | 0.781 | -0.237 | -0.855* | 0.959** | 0.240 |
| SUVA | -0.580 | -0.005 | 0.872* | -0.902* | 0.189 |
| pH | -0.417 | 0.868* | 0.008 | 0.181 | -0.847* |
| NH**_4_-**N | 0.439 | -0.617 | -0.362 | 0.042 | 0.941** |
| NO**_3_-**N | -0.573 | 0.703 | 0.514 | -0.179 | -0.891* |
| Organic nitrogen | 0.816* | -0.332 | -0.730 | 0.938** | 0.238 |
| Total phosphorus | 0.429 | -0.865* | 0.181 | -0.146 | 0.781 |
| Total iron | 0.346 | -0.829* | 0.101 | -0.258 | 0.867* |
| Chlorophyll *a* | 0.926** | -0.884* | -0.58 | 0.616 | 0.537 |
| Total seston | 0.852* | -0.955** | -0.333 | 0.439 | 0.621 |
| DISTANCE | 0.820* | -0.829* | -0.506 | 0.517 | 0.403 |

Table S6d. Correlation coefficients (*p<0.05, **p<0.01) between Werenskiold Glacier cryoconite holes chosen biological parameters and non-biological factors. TC – total microbial count, PHAC/TC – photoautotrophic cell count to total microbial count ratio, DGGE – taxonomical diversity of samples by taxonomic operational units (OTU’s), DISTANCE – distance from glacier edge, CFU/TC– colony forming units on R2A agar to total microbial count ratio, %Cyan - percentage contribution of cyanobacterial cells to photoautotrophic count, DOC – dissolved organic carbon, TOC – total organic carbon, SUVA – aromaticity content of dissolved organic carbon.

| **WC samples** | TC | CFU/TC | %Cyan | DGGE | DISTANCE |
| --- | --- | --- | --- | --- | --- |
| TC | 1.000 | 0.574 | 0.972** | 0.741 | 0,090 |
| PHAC/TC | 0.601 | 0.047 | 0.570 | 0.571 | -0,653 |
| %Cyan | 0.972** | 0.502 | 1.000 | 0.597 | 0,000 |
| DOC | -0.309 | -0.850* | -0.191 | -0.553 | -0,913* |
| TOC | -0,265 | -0,853* | -0,136 | -0,567 | -0,893* |
| SUVA | 0.402 | 0.819* | 0.456 | 0.319 | 0,605 |
| NH**_4_-**N | -0.887* | -0.870* | -0.832* | -0.804 | -0,470 |
| PO_4_-P | -0.749 | -0.764 | -0.599 | -0.832* | -0,616 |
